# Bioluminescence for in vivo detection of cell-type-specific inflammation in a mouse model of uveitis

**DOI:** 10.1038/s41598-020-68227-4

**Published:** 2020-07-09

**Authors:** Sarah John, Kevin Rolnick, Leslie Wilson, Silishia Wong, Russell N. Van Gelder, Kathryn L. Pepple

**Affiliations:** 1 Department of Ophthalmology, University of Washington, Seattle, WA 98104 USA; 2 Department of Biological Structure, University of Washington, Seattle, WA 98195 USA; 3 Department of Pathology, University of Washington, Seattle, WA 98195 USA

**Keywords:** Imaging the immune system, Experimental models of disease, Animal disease models, Inflammation, Uveal diseases

## Abstract

This study reports the use of cell-type-specific in vivo bioluminescence to measure intraocular immune cell population dynamics during the course of inflammation in a mouse model of uveitis. Transgenic lines expressing luciferase in inflammatory cell subsets (myeloid cells, T cells, and B cells) were generated and ocular bioluminescence was measured serially for 35 days following uveitis induction. Ocular leukocyte populations were identified using flow cytometry and compared to the ocular bioluminescence profile. Acute inflammation is neutrophilic (75% of ocular CD45 + cells) which is reflected by a significant increase in ocular bioluminescence in one myeloid reporter line on day 2. By day 7, the ocular T cell population increases to 50% of CD45 + cells, leading to a significant increase in ocular bioluminescence in the T cell reporter line. While initially negligible (< 1% of CD45 + cells), the ocular B cell population increases to > 4% by day 35. This change is reflected by a significant increase in the ocular bioluminescence of the B cell reporter line starting on day 28. Our data demonstrates that cell-type-specific in vivo bioluminescence accurately detects changes in multiple intraocular immune cell populations over time in experimental uveitis. This assay could also be useful in other inflammatory disease models.

## Introduction

Post mortem assays such as histology, immunohistochemistry, and flow cytometry are the current gold standard assays for identifying immune cells in eyes with experimental uveitis^1–3^. While these are powerful tools and can provide high resolution information about the infiltrating cell populations, they are all terminal assays. In vivo imaging modalities provide biomarkers that can be assessed longitudinally, thereby reducing the number of animals required for well-powered experiments, as well as allowing for more detailed time courses of disease than are feasible with terminal experiments. Previously, we demonstrated that in vivo imaging techniques such as optical coherence tomography (OCT) and luminol based bioluminescence imaging can be used for quantitative longitudinal analysis in animal models of uveitis^4,5^. However, the former does not distinguish between infiltrating immune cells, and the latter is limited to study of myeloid cells expressing myeloperoxidase^6^. We therefore sought to develop a more generalized method for measuring and characterizing intraocular inflammation in murine models.

In vivo bioluminescence imaging (BLi) has become a widely used tool for studying biological processes in small laboratory animals^7–9^. BLi requires the generation of photons from within the tissue of interest, and a method for capturing and processing these photons using a charge coupled device (CCD) camera^10^. In vivo bioluminescence is typically engineered using the combination of a transgene expressing firefly luciferase and exogenous administration of luciferin prior to imaging^11^. In contrast to another common in vivo imaging strategy using fluorescent proteins such as green fluorescent protein (GFP), BLi does not require an external source of illumination for fluorochrome excitation. This allows BLi to avoid the challenges of endogenous autofluorescence and the risk of light induced tissue damage that can limit in vivo fluorescence imaging strategies.

Prior applications of BLi in animal models of ocular disease include tracking the extent of herpes simplex virus type 1 (HSV-1) systemic spread after corneal infection, tracking luciferase expressing neoplasm growth and response to therapy, and following gene expression in a model of intra-ocular gene therapy^12,13,14^. In this study we test the feasibility of using immune cell-type-specific BLi to detect changes in the intraocular leukocyte populations in the primed mycobacterial uveitis (PMU) mouse model, and then compare BLI results to gold standard post-mortem assay of flow cytometry.

## Results

### Generation and validation of four transgenic mouse lines for inflammatory cell specific bioluminescence

**Figure 1:**
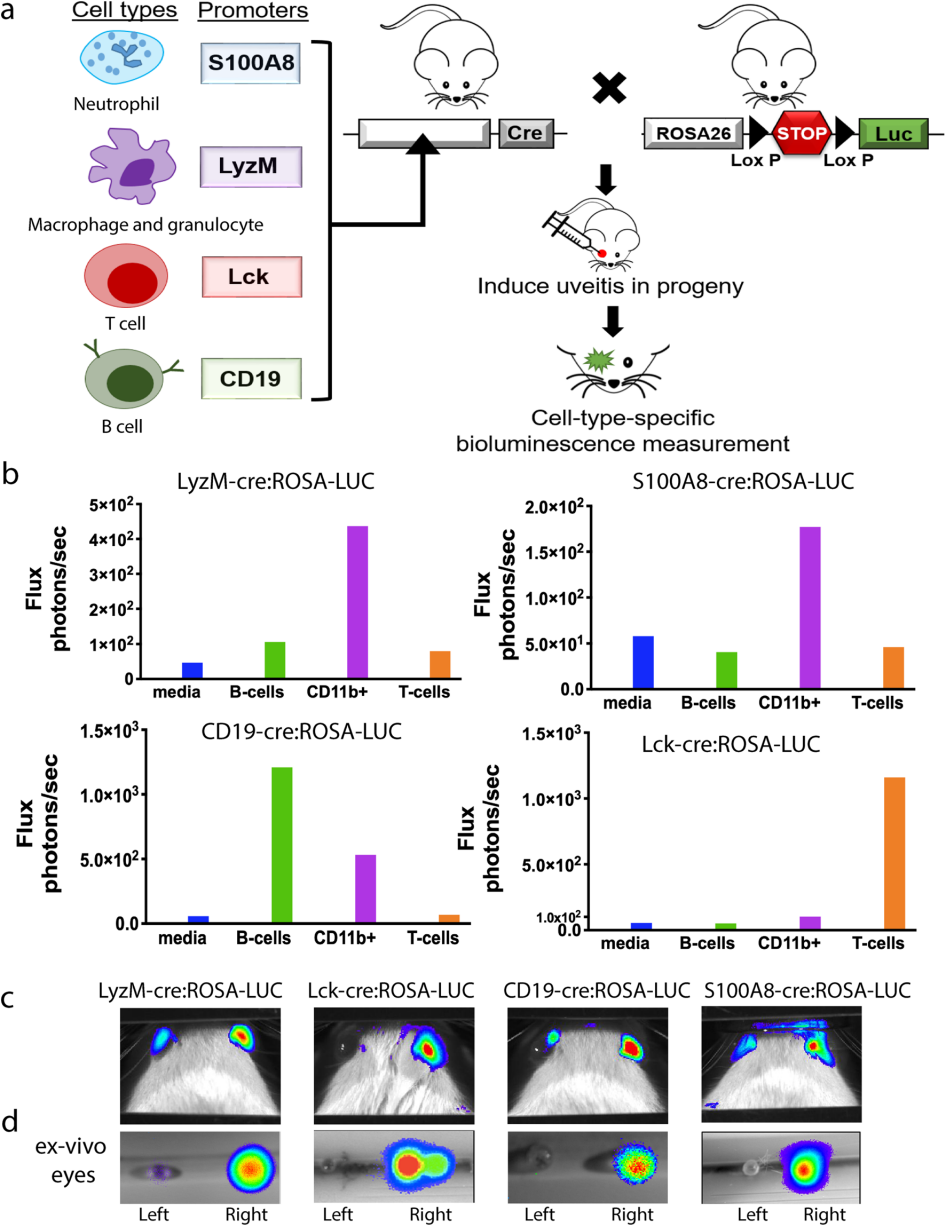
Four luciferase reporter lines generate immune cell-type-specific luciferase expression. (**a**) Schematic of the overall experimental strategy. (**b**) In vitro quantification of splenocyte bioluminescence from each of the reporter strains. From each reporter strain, splenocytes were sorted into the T cell or B cell population by negative selection or the CD11b + population using positive selection. 10^6^ sorted cells were incubated in DMEM media with Luciferin and bioluminescence recorded with a luminometer. (**c**) Uveitis was induced in the right eye of each luciferase reporter line, and BLi performed on day 2 for LyzM-cre:ROSA-LUC and S100A8-cre:ROSA-Luc animals,day 7 for Lck-cre:ROSA-LUC and day 21 for CD19-cre:ROSA-LUC. A colorized photon density map is overlaid on the black and white image of the mouse head. The area of highest photon density in the image is indicated in red and overlays the right eye in all images. Flux from the right eye of each animal are as follows: LyzM-cre:ROSA-LUC 1.1 × 10^7^ p/s, Lck-cre:ROSA-LUC 2.5 × 10^5^ p/s, CD19-cre:ROSA-LUC animals 8.1 × 10^5^ p/s, and S100A8-cre:ROSA-Luc 8.1 × 10^6^ p/s. (**d**) Ex vivo BLi of the left and right eyes enucleated from the animals shown in panel B. A colorized photon density map is overlaid on the black and white image of the enucleated eyes. Extraocular tissue was carefully removed from both eyes prior to imaging.

Mice expressing luciferase in separate inflammatory cell populations were generated using a ROSA26-Luciferase (ROSA-LUC) transgene preceded by a floxed transcriptional stop sequence^15^. When crossed to a cell-type-specific Cre-recombinase expressing line, the stop sequence is removed, and luciferase is constitutively expressed. Luciferase gene expression was targeted to neutrophils with S100A8-Cre, myeloid cell lineages with LyzM-cre, T-cells with Lck-cre, and B cells with Cd19-cre (Fig. 1a)^16–18^.

Successful generation of the cell-type-specific luciferase expressing populations was verified by testing magnetically sorted splenocyte populations (T cells, B cells and CD11b + myeloid cells) for in vitro bioluminescence (Fig. 1b). From LyzM-cre:ROSA-LUC and S100A8-cre:ROSA-LUC animals, only CD11b + splenocytes demonstrated in vitro bioluminescence. Conversely, from LCK-cre:ROSA-LUC animals, only the negatively selected T cell fraction produced bioluminescence. From CD19-cre:ROSA-LUC animals, the majority of bioluminescence was produced by the negatively selected B cell fraction. However, some bioluminescence was also noted in the positively selected CD11b + fraction. This is consistent with a known population of CD11b + B-cells in the spleen, the so called B1 population^19^.

### In vivo detection of intraocular bioluminescent inflammatory cells in the PMU model

To determine if in vivo bioluminescence could be used to detect inflammation in eyes with uveitis, we induced PMU in the right eye of one animal from each cre:ROSA-LUC line and evaluated both eyes for the presence of bioluminescence (Fig. 1c). Prior to BLi, clinical inflammation in the right (injected) eye was confirmed and scored using OCT. No clinical inflammation was seen in any left (uninjected) eye. For each cre:ROSA-LUC line, the photon density map indicates the presence of high photon flux (red) from the injected eye. In the control (left) eye of the LyzM-cre:ROSA-LUC, S100A8-cre:ROSA-LUC, and CD-19:ROSA-LUC lines an additional low density photon flux (blue/green) signal is also present. Since none of the fellow eyes demonstrated clinical inflammation by OCT, we hypothesized that the fellow eye bioluminescence could be produced from a population of leukocytes present in normal periocular tissue^20^. To test this possibility, we enucleated both eyes, carefully dissected away the periocular tissues including the conjunctiva, and repeated the BLi on the ex vivo globes (Fig. 1d). Post enucleation, bioluminescence is only detected in the inflamed (right) eye, consistent with low-level in vivo bioluminescence produced by periocular tissue.

### Longitudinal flow cytometry characterization of intraocular leukocytes in PMU

**Figure 2:**
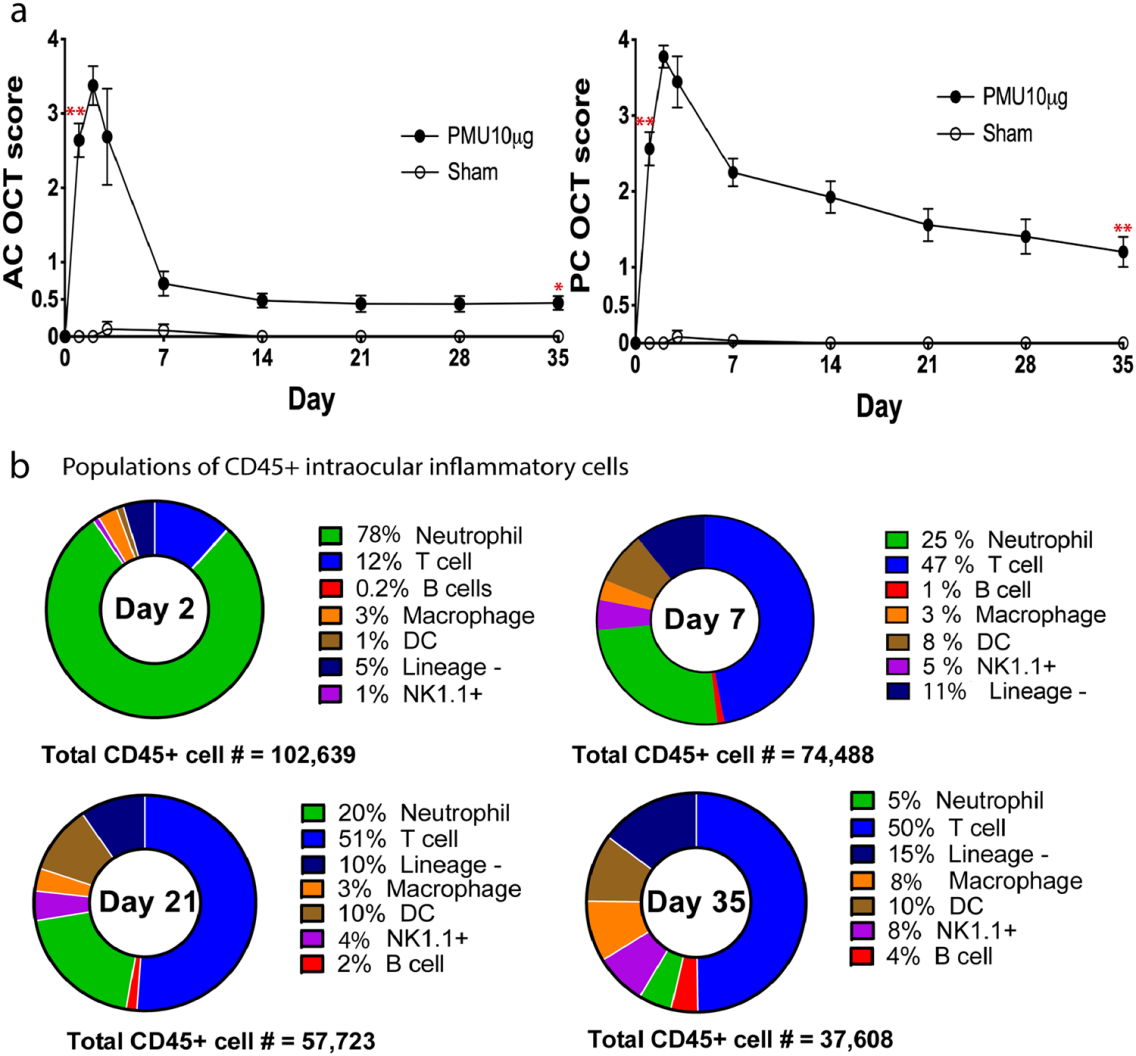
Longitudinal OCT clinical score and flow cytometry characterization of PMU eyes. OCT score of the (**a**) anterior chamber (AC) and posterior chamber (PC) for PMU (filled circles) and sham (open circles) injected eyes at baseline and days 1, 2, 3, 7, 14, 21, 28, and 35 after initiation of uveitis. The average score shown is for all animals used for both BLi and flow cytometry (PMU, n = 108; sham, n = 9). error bar indicates SEM. Difference between PMU and Sham scores on day 2 and day 35 tested using the Mann Whitney U-test, *p < 0.01, **p < 0.001. (**b**) Pie charts depict the cell populations identified in the inflamed PMU eye as a % of total CD45 + cells. Percentages from each day are the average of three independent experiments. See methods for cell surface markers used to define specific lineages. The total number of CD45 + cells analyzed to generate the percentages are shown for each day.

Primed mycobacterial uveitis (PMU) is a model of post-infectious uveitis that is generated by combined systemic and intravitreal injection(IVT) of mycobacterial antigens^21–28^. In this model, only the right eye is injected with mycobacterial antigens. The left eye is untreated and functions as an internal uninflamed control eye. In injected (right) eyes, anterior chamber (AC) and posterior chamber (PC) inflammation scores peaked 2 days after injection, decreased daily through day 7, and then established low level chronic inflammation that continued through day 35 (Fig. 2a). Sham injection with PBS instead of mycobacterial antigen does not generate uveitis (open circles in Fig. 2a), and inflammation scores of PMU injected eyes on day 1 were significantly higher than Sham injected eyes in the AC (PMU score, 2.6 ± 1.3, Sham score, 0; p < 0.001) and PC (PMU score, 2.6 ± 1.2, Sham score, 0; p < 0.001). On day 35, mean PMU AC score (0.5 ± 1.3) and PC score (1.2 ± 1.1) have decreased compared to day 1, but are still significantly higher than sham injected eyes (p = 0.002, p < 0.001).

To establish the populations of leukocytes infiltrating inflamed PMU eyes, flow cytometry was performed on days 2, 7, 21, and 35 after intravitreal injection (IVT) (Fig. 2b). Major leukocyte lineages were identified, and the population sizes determined as a percentage of the total intraocular CD45 + population. The results shown at each time point in Fig. 2b are the average population percentages determined from 3 independent experiments.

During acute inflammation (day 2 after IVT), the CD45 + infiltrate primarily consisted of neutrophils (78%) and T cells (12%) (Fig. 2b). The remaining 10% of cells were a combination of macrophages (3%), dendritic (DC) cells (1%), natural killer (NK) cells (1%), and lineage-negative cells (5%). At this time point B cells (0.2%) were rare. By day 7, the neutrophil population decreased to 25% and T cells increased to 47% of the CD45 + population. Other myeloid populations constituted an additional 11% of the CD45 + cells including 8% dendritic cells and 3% macrophages at this subacute timepoint. In contrast, the CD19-positive B cell population only increased slightly to 1% of the CD45 + cells. On each subsequent day evaluated by flow (Days 21 and 35) the contribution of myeloid cells to the intraocular CD45 + population declined and the contribution of lymphocyte cells increased. By day 35, neutrophils decreased to 5% of the CD45 + population, while T cells remained 50%, and B-cells increased to 4% of the total intraocular CD45 + population.

### Characterization of the natural history of uveitis in PMU using in vivo immune cell bioluminescence

**Figure 3:**
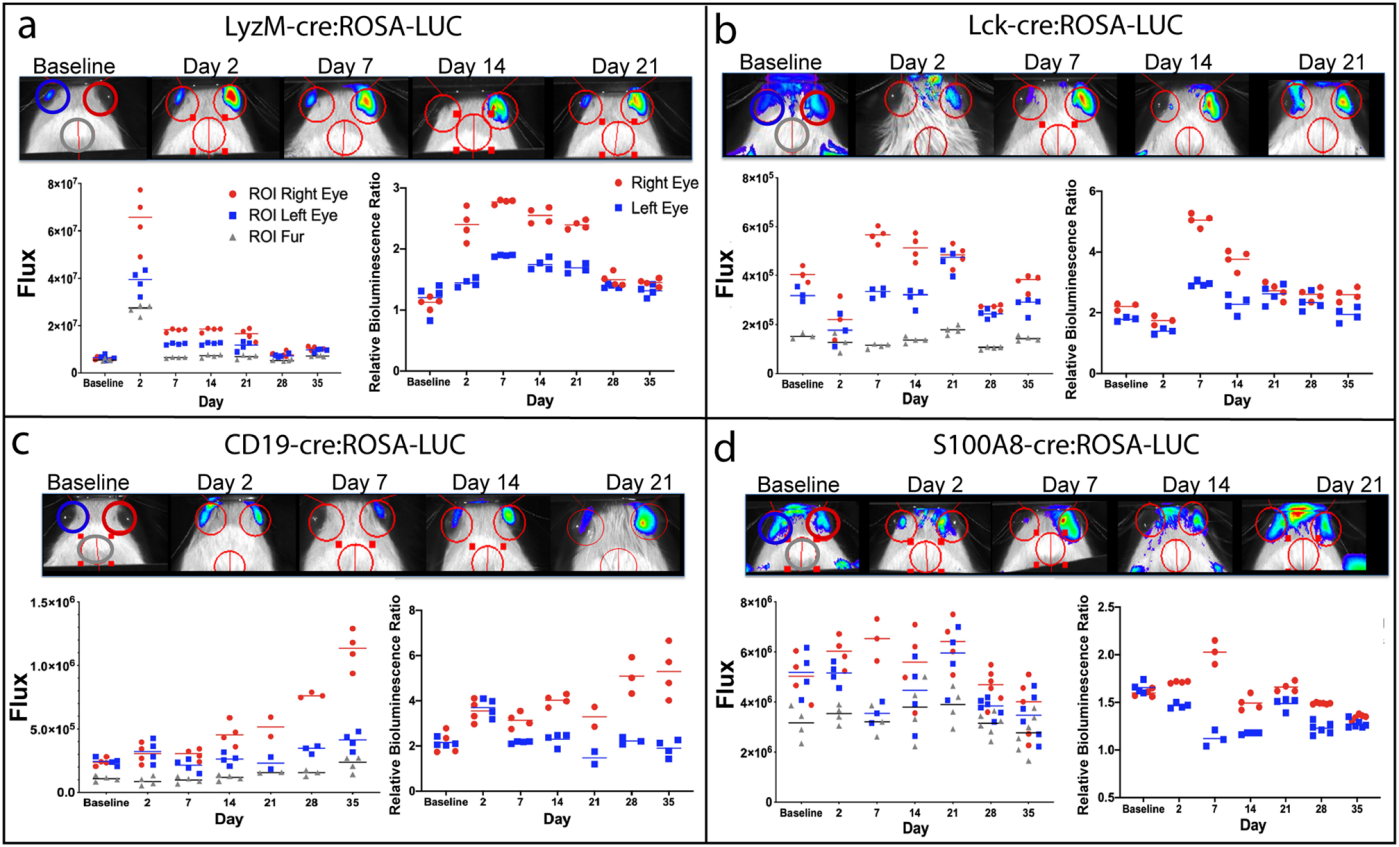
Longitudinal quantification of bioluminescence in the PMU model. For each cre:ROSA-LUC line, individual animals were imaged prior to uveitis induction (baseline) and then longitudinally. One animal of each bioluminescent line is shown in panels a-d. The photon density map and ROIs are overlaid on the black and white image of the mouse on each day. The flux (photons/s) through each ROI on each day is displayed in the lower left graph (Right eye = red circles, left eye = blue squares, fur = grey triangles). The graph on the lower right displays the relative bioluminescence ratio (RBR = eye ROI flux/fur ROI flux) for the right eye (red circles) or left eye (blue squares) for each day. (**a**) Myeloid reporter LyzM-cre:ROSA-LUC (**b**) T cell reporter Lck-cre:ROSA-LUC (**c**) B cell reporter CD19-cre:ROSA-LUC (**d**) Neutrophil reporter S100A8-cre:ROSA-LUC.

Next we tested the ability of in vivo bioluminescence from the cre:ROSA-LUC lines to detect the changes in the inflammatory cell populations in PMU eyes (Fig. 3). Results of one animal from each cre:ROSA-LUC line are shown in Fig. 3, panels a-d. For each animal, bioluminescence in flux (photons/second) was measured at baseline, day 2, day 7 and then weekly through day 35 following uveitis induction. On each image, three regions of interest (ROIs) were identified; the left or control eye (circled in blue), the inflamed eye (circled in right), and the top of the head which was used to establish background bioluminescence (circled in gray). One each day, flux was measured (3–4) times for each ROI and the mean assigned as the final ROI flux. Repeated measurements of each ROI are depicted by the multiple squares, circles, or triangles at each timepoint. The repeated measurements of flux on all days were also used to determine the mean coefficient of variation (mean CV) for each line. The mean coefficient of variation of the inflamed eye ROI was 15.38% in LyzM-cre:ROSA-LUC mice, 13.70% in Lck-cre:ROSA-LUC mice, 16.75% in CD19-cre:ROSA-LUC mice and 19.5% in S100A8-cre:ROSA-LUC mice.

Flux from each eye ROI was also converted into the relative bioluminescence ratio (RBR) at each time point. The RBR is calculated by dividing the flux of the eye by the background flux (from the fur ROI). The RBR normalizes the flux values of the eye ROIs to the background bioluminescence to facilitate comparisons across subsequent days. Separation between the inflamed eye ROI (in red) and the control eye ROI (in blue) highlights the differential bioluminescence attributed to inflammatory cell infiltration in the eye with uveitis. Different patterns of bioluminescence associated with ocular inflammation are generated over time in each line.

In the LyzM-cre:ROSA-LUC animal (Fig. 3a), flux in the right eye increased by one log from 6.2 × 10^6^ p/s at baseline to 6.5 × 10^7^ p/s on day 2 after intravitreal injection. Flux in the left eye also increased from 8.6 × 10^6^ p/s at baseline to 3.9 × 10^7^ p/s on day 2, but the increase was less than in the right eye. Of note, the background flux also increased from 5.1 × 10^6^ p/s at baseline to 2.7 × 10^7^ p/s on day 2. To normalize the contribution of this increase in background flux to the ocular flux, the RBR for each eye ROI was calculated. The RBR highlights the differential increase in flux in the right eye (2.4) when compared to the left eye (1.5). This pattern of higher bioluminescence in the right eye began on day 2 and continued through day 28 when total flux returned to baseline levels in both eyes and the difference between right and left RBR resolved. In the LCK-cre:ROSA-LUC animal (Fig. 3b), right eye flux increased on day 7 flux in the inflamed eye from 4.1 × 10^5^ p/s at baseline to 5.7 × 10^5^ p/s. This increase was also reflected in the higher RBR of the inflamed eye (5.1) compared to the fellow eye (2.9) that persisted until day 21. In the CD-19-cre:ROSA-LUC animal (Fig. 3c), flux in the inflamed eye did not increase above baseline levels (3.5 × 10^5^ photons/s) until day 14 (4.3 × 10^5^ photons/s). All subsequent measurements showed an increased flux and RBR in the inflamed eye compared to the fellow eye with a maximum mean flux on day 35 of 1.1 × 10^6^ photons/s. The inflamed eye of the S100A8-cre:ROSA-LUC animal (Fig. 3d) showed slightly higher bioluminescence on day 2 (6.0 × 10^6^ photons/s) when compared to baseline (5.0 × 10^6^ photons/s), and the RBR in the right eye was differentially elevated compared to the left eye. The difference between right and left eye ROIs was maximal on day 7, but overall the differential between eyes in the S100A8-cre:ROSA-LUC animal was the least impressive among the bioluminescent animals.

**Figure 4:**
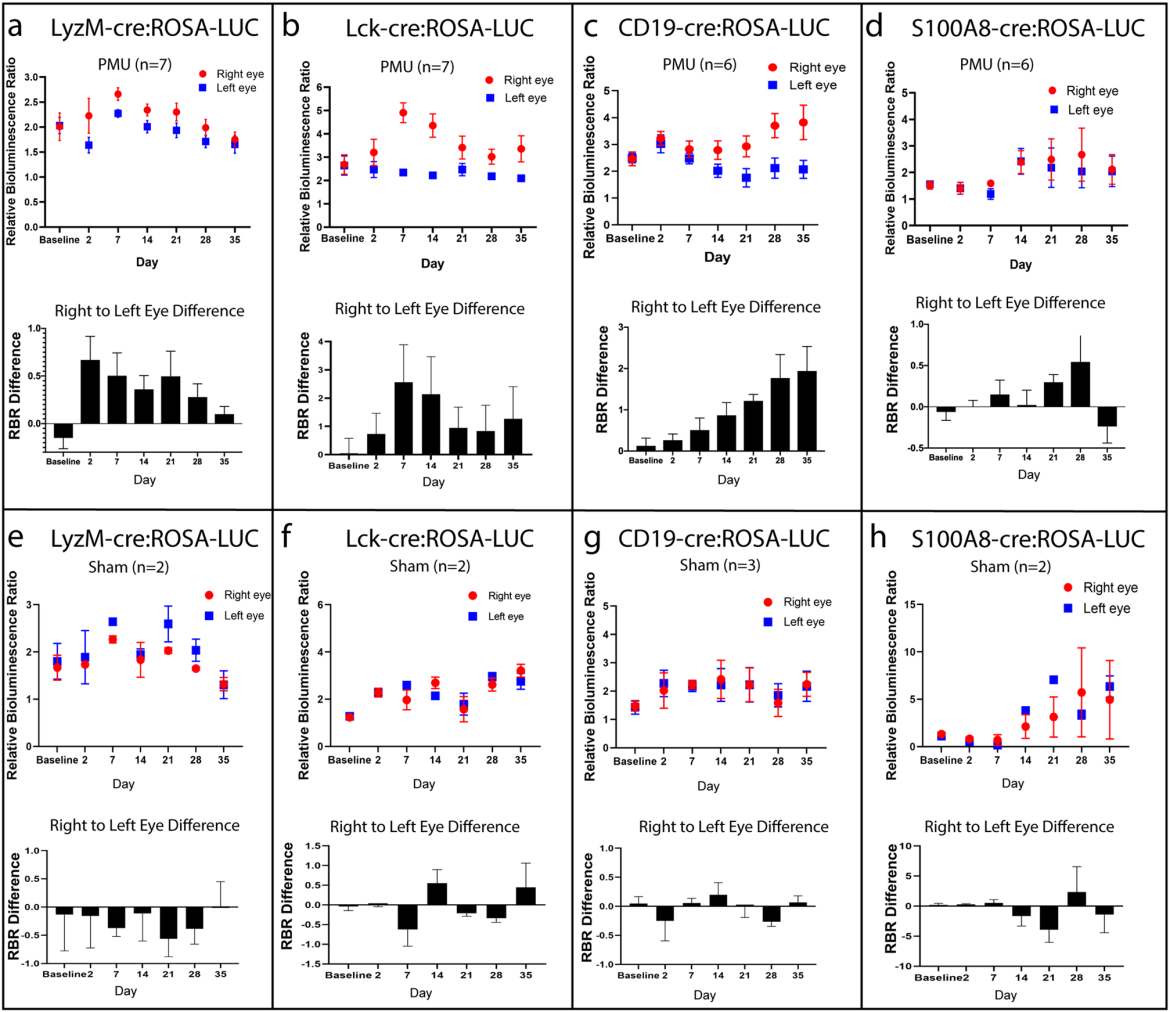
Longitudinal bioluminescence identifies changes in myeloid, T cell, and B cell populations in eyes with PMU. In each cre-ROSA:LUC line, bioluminescence was measured longitudinally in multiple animals treated with PMU injection in the right eye (**a**–**d**) or sham injection (**e**–**h**). Average relative bioluminescence ratios (RBRs) of the injected right (red circle) and fellow left (blue square) eyes were calculated. The number of animals represented in each panel are indicated (n = #). (**a**,**e**) Myeloid reporter LyzM-cre:ROSA-LUC (**b**,**f**) T cell reporter Lck-cre:ROSA-LUC (**c**,**g**) B cell reporter CD19-cre:ROSA-LUC (**d**,**h**) Neutrophil reporter S100A8-cre:ROSA-LUC. The top graph shows the average RBR on each day. The bottom graph shows the difference between right and left RBR on each day. Error bars = SEM. For flux data used to calculate the RBR in this figure see supplementary Fig. 3.

Figure 4 shows the results of longitudinal cell-type-specific bioluminescence measured in a total of 35 animals that were tested in two experimental conditions. First, as previously described, bioluminescence was measured in a cohort of animals following intravitreal injection of mycobacterial extract into the right eye (PMU) (Fig. 4a–d). A second cohort of animals received intravitreal injection of PBS to the right eye (sham injection controls) (Fig. 4e–h). The results of all PMU animals (n = 6–7) or sham injected controls (n = 2–3) from each cre:ROSA-LUC line were used to generate the inflamed cell-type-specific longitudinal bioluminescence profiles shown in Fig. 4 and supplementary Fig. 3. The average RBR on each day is presented in Fig. 4 with the injected eyes shown in red and uninjected fellow eyes in blue. The bottom graph in each panel is the average difference between the right and left eye RBR on each day (black bar). Flux data used to calculate the RBR is shown in supplementary Fig. 3.

Average bioluminescence obtained in the cohorts of PMU treated animals from each of the cre:ROSA-LUC lines was similar to that of the individual animals presented in Fig. 3 with the exception of S100A8-cre:ROSA-LUC. Shown in Fig. 4a, the myeloid line LyzM-cre:ROSA-LUC cohort shows differential bioluminescence on days 2–28 with a maximum difference between injected and fellow eyes on day 2. By day 35, the difference in bioluminescence between the right and left eyes was resolved. Differential expression in the T cell line Lck-cre:ROSA-LUC (Fig. 4b) also begins on day 2. However, the maximum difference between injected and fellow eyes does not develop until day 7, and differential expression continues through day 35. In the B cell line CD19-cre:ROSA-LUC, differential expression does not begin until day 14, but then the difference between injected and fellow eyes continues to increase though day 35 (Fig. 4c). In contrast to the other three lines, differential expression was not demonstrated in the S100A8-cre:ROSA-LUC cohort (Fig. 4d).

The control cohort of animals treated with Sham injection did not exhibit elevated RBR of the right eye compared to the left eye or a similar pattern of differential bioluminescence over the 35-day time course (Fig. 4e–h, and supplementary Fig. 3).

Finally, we compared the bioluminescence profiles associated with myeloid cells, T cells and B cells to the flow cytometry data to determine if cell-type-specific bioluminescence has the ability to accurately reflect immune cell population dynamics in eyes with uveitis. Flow cytometry analysis established that the acute phase of PMU is dominated by myeloid populations like neutrophils (CD45 + , CD11b + , Ly6G +) and inflammatory macrophages (CD45 + , CD11b + , Ly6G-, Ly6C +) (Fig. 2b). We would therefore expect the CD11b + myeloid cells responsible for luciferase expression in the LyzM-cre:ROSA-LUC and S100A8-cre:ROSA-LUC lines to have a bioluminescence profile that peaks early in the time course. As shown in Fig. 4a, the myeloid influx is reflected in the LyzM-cre:ROSA-LUC line by the elevated RBR difference in the right eye compared to the left eye starting on day 2. In contrast, the S100A8-cre:ROSA-LUC profile did not show differential bioluminescence in the right eye during acute timepoints (Fig. 4d), but later in the time course (day 28) there was a suggestion of differential expression in PMU treated right eyes. However, this differential was also seen in the sham injection animals suggesting that the differential bioluminescence noted on day 28 was not specific for uveitis associated inflammation.

Flow cytometry identified a substantial increase in the CD3 + T cell population in PMU eyes between day 2 (12% of CD45 + cells) and day 7 (47% of CD45 + cells). This was reflected in the bioluminescence profile by the maximum right to left eye RBR difference on day 7 in the Lck-cre:ROSA-LUC animals (Fig. 4b). Flow also showed that while T cells continued as the major leukocyte population in the eye, total infiltrating cells decreased by nearly half between days 7 and 35. This decrease in T cell number is also reflected by the decrease in RBR differential between days 7 and 35 in the Lck-cre:ROSA-LUC animals. However, unlike the LyzM-Cre:ROSA-LUC bioluminescence, right eyes of PMU treated Lck-cre:ROSA-LUC animals continue to produce more bioluminescence than fellow left eyes though day 35.

Finally, B cell population dynamics were compared. Flow analysis determined that the CD19 + B cell contribution to intraocular PMU inflammation was minimal at early timepoints (0.02% of CD45 + cells on day 2), but progressively increased over time to 4% of CD45 + cells by day 35 (Fig. 2b). BLi first detected an increase in the B cell population in the eye on day 14 when flow determined they constituted between 1% (day 7) and 2% (day 21) of CD45 + cells. Bioluminescence continued to reflect the flow data with an increasing RBR differential in PMU treated animals through day 35 in the CD19-cre:ROSA-LUC animals (Fig. 4c).

## Discussion

In this study, we use in vivo bioluminescence to track the changes in intraocular leukocyte populations in the PMU mouse model. When compared to the population dynamics defined by flow cytometry, the bioluminescence profiles for LyzM-cre:ROSA-LUC, Lck-cre:ROSA-LUC, and CD-19-cre:ROSA-LUC recapitulate key aspects of the PMU model. First, the acute myeloid response was reflected by the peak of LyzM-cre:ROSA-LUC bioluminescence seen on day 2 followed by a return to baseline levels as the myeloid population declined. Second, the increase in the T cell population from day 1 to day day 7 is accurately reported by the peak of bioluminescence in Lck-cre:ROSA-LUC on day 7, and the chronic presence of a large number of T cells in the eye is reflected by continued right eye bioluminescence through day 35. Finally, bioluminescence from inflamed CD19-cre:ROSA-LUC eyes reflects the gradual but steady increase in the B cell population from < 1% on day 1 to > 4% on day 35.

All lines except the S100A8-cre:ROSA-LUC line demonstrated patterns of bioluminescence consistent with the flow data. Due to expression of S100A8 in neutrophils and the large neutrophil population identified in PMU eyes during acute inflammation, we anticipated that bioluminescence from the S100A8-cre:ROSA-LUC would be strong and specific to the inflamed eye starting on day 2. However, the bioluminescence pattern generated by S100A8-cre:ROSA-LUC did not perform as expected. While ex vivo data did verify that this line can generate intraocular bioluminescence and that it is specific to the inflamed eye, this differential bioluminescence was not reliably detected in vivo. We did not perform additional experiments to clarify why this line failed to demonstrate an inflammation dependent signal. However, we can propose some potential mechanisms. One possible explanation could be that luciferase function is compromised when it is co-expressed in cells such as neutrophils that also produce toxic inflammatory mediators upon activation. The half life and stability of the luciferase enzyme is impacted by a number of factors including reactive oxygen species (ROS) such as H_2_O_2_^29,30^. Activated neutrophils generate peroxide as part of the oxidative burst, and express proteases such as serine proteases, cathepsin G, neutrophil elastase and proteinase 3^31^. Taken together, it seems likely that the activated neutrophils infiltrating PMU eyes offer a sub-optimal intracellular environment for luciferase function. An alternative mechanism for our failure to detect the differential bioluminescence could be the result of S100A8 expression in cells other than neutrophils, such as in keratinocytes of the epidermis^32^. While imaging the S100A8-cre:ROSA-LUC animals, we did notice that exposed skin of the ears, paws, tail, and snout exhibited bioluminescence prior to any intervention. While this could represent bioluminescence from intravascular neutrophils, it could also be a signal from non-lymphoid cells in the skin. Thus, we may have failed to detect PMU inflammation with this line due to the presence of widespread skin bioluminescence that rendered the signal coming from the eye insufficient to detect. Regardless of the ultimate mechanism, this line did not provide a reliable in vivo reporter for the myeloid contribution to intraocular inflammation in PMU. We would recommend that future applications of this BLi technique focus on the LyzM-cre:ROSA-LUC line for myeloid cell detection and monitoring.

While this study demonstrates that immune cell-type-specific bioluminescence can reliably detect time-dependent changes in intraocular immune cell population in vivo, there are limitations to this strategy. The high background bioluminescence in most of the lines generated an overall modest signal-to-noise ratio associated with inflammation. Due to the presence of immune cells throughout the body, this background bioluminescence is not inherently surprising. However, it may limit reliable detection of inflammation that is mild or located in deeper tissues. As an alternative approach, immune cells from these transgenic animals may be easier to detect at sites of inflammation if they were adoptively transferred into animals lacking luciferase expression. The animal model of uveitis Experimental Autoimmune Uveitis (EAU) has a well established adoptive transfer method for generating uveitis^33,34^. By transferring uveitogenic T cells generated in the bioluminescent Lck-cre:ROSA-LUC line into animals lacking the luciferase transgene, the background levels of bioluminescence would only be orders of magnitude lower (around 4 × 10^3^ photons/s, see supplementary Fig. 1). This strategy could potentially provide a higher signal to noise ratio for use in detecting the luciferase expressing cells and quantifying their contribution to uveitis in the EAU model using in vivo bioluminescence.

Ultimately, the power of an immune cell-type-specific bioluminescence assay would be to deliver a quantitative determination of the number of cells present in the eye. The goal of this pilot study was to evaluate the longitudinal bioluminescence profile generated by each transgenic line, and animals were not sacrificed during their time course to perform flow analysis. Therefore, the current study does not allow direct point-by-point comparison of intraocular cell number and total ocular bioluminescence in individual animals. Future studies will be needed to fully validate the quantitative aspect of immune cell specific bioluminescence. Specifically, average flux per cell could be estimated by measuring in vivo bioluminescence in the uveitis model and then collecting individual eyes to determine the absolute number of immune cells present in each eye. However, collecting and counting every immune cell from an individual eye is not technically feasible, so these results would at best be estimates for absolute photon flux per cell. Living and lysed splenocytes could also be used in serial dilutions to more precisely measure the average flux per cell in each cell type under optimal in vitro conditions. Ultimately it may prove challenging to use in vivo flux to accurately quantify the absolute number of inflammatory cells in an inflamed tissue. However, since this assay can be performed repeatedly in the same cohort of animals, relative quantitation may be sufficient for many applications.

Due to the small population of CD19 positive cells identified by flow cytometry, the intensity of the bioluminescent signal from the B-cell reporter was an unanticipated finding. One possible explanation is that the relative paucity of B cells in the native eye provides a high signal to noise ratio for detecting inflammation with this transgenic line. Another possibility is that a plasma cell population with decreased or no expression of CD19 develops in the eye during the chronic inflammatory phase of PMU^35,36^. These plasma cells would not be detected by flow cytometry utilizing anti-CD19, but would still express luciferase due to the permanent removal of the stop signal after CD19-cre mediated recombination. The role of B cells in human disease and animal models of uveitis is not well studied or understood^37^, but recent reports have identified ectopic lymphoid structures that are rich in B cells in both human and animal models of EAU^38–41^. Our data suggests that the CD19-cre:ROSA-LUC could be a useful tool for studying B cells dynamics in the EAU model. Furthermore the strong bioluminescence signal from PMU CD19-cre:ROSA-LUC eyes suggests that B cells play a more general role in uveitis than previously appreciated since PMU and EAU are mechanistically distinct forms of EAU^2^. Further studies to understand the role of B cells, and possibly plasma cells, in the mechanisms of PMU are warranted.

One limitation of this assay is the within-animal variation in background bioluminescence that was noted sporadically over the course of the study. To date, we have not identified a single variable responsible for these episodes. To minimize variation, we recommend standardization of the intraperitoneal injection protocol (location, depth, weight-based dose), precise timing of the interval between intraperitoneal injection of luciferin and the beginning of the imaging session, batch luciferin preparation and storage of individual use aliquots. By using the PMU model, both an inflamed and a control eye are present which allows the calculation of the "relative bioluminescence ratio" to minimize the impact of these sporadic baseline changes on the ability to detect differential or increased bioluminescence in the inflamed eye. This fellow eye control may not be available in all animal models. An additional limitation of the BLi approach is that it does not have sufficient resolution to discriminate between bioluminescence produced by intraocular inflammation from bioluminescence produced by combined intraocular and periocular inflammation. Therefore, any signs of lid or orbital inflammation should be noted and considered as possible confounders contributing to bioluminescence within the ocular ROI. Some additional limitations and considerations include that choroidal pigment may attenuate bioluminescence from intraocular structures in non-albino animals, specific transgenic/knock-in strains are needed to generate bioluminescence, and the size limitations of currently available imaging devices limits application at present to mice.

Finally, we have not considered what impact different functional states or maturation states could have on cell type specific bioluminescence. For example, bioluminescence from the LyzM-cre:ROSA-LUC line was highest in the acute stages of PMU inflammation and decreased over time. We have concluded that this is consistent with the flow data identifying a decrease in the total number of macrophages in the eye. However, as an alternative explanation, it is possible that bioluminescence could be different in activated macrophages and epithelioid giant cells in chronic granulomas. It is also possible that memory T cells or plasma cells could have different capacity for bioluminescence than acutely activated T and B cells. These are all interesting possibilities that warrant additional exploration.

In summary, our results suggest that in vivo immune-cell-specific bioluminescence is a useful technique for understanding the temporal development of immune cell infiltrates in a rodent model of uveitis. This technique should be applicable to other models of uveitis and ocular inflammatory diseases, and could be used to follow the immune responses associated with other insults to the eye such as light-mediated retinal degeneration, glaucomatous retinal ganglion cell degeneration, diabetic retinopathy, or gene therapy induced uveitis.

## Methods

### Animals

The animal study protocol was approved by the Animal Care and Use Committee of the University of Washington (animal study protocol #4481-02) and was compliant with ARVO guidelines for use of animals in vision research. Transgenic mice that express luciferase in specific inflammatory cell lines were generated using a Cre-loxP strategy. Four cre recombinase expressing strains, B6.Cg-Tg(S100A8-cre,-EGFP)1Ilw/J (JAX stock #021614; referred to as S100A8-cre), B6.129P2(C)- Cd19tm1(cre)Cgn/J (JAX stock #006785; referred to as Cd19-cre), B6.Cg-Tg(Lck-cre)548Jxm/J, (JAX stock #003802; referred to as Lck-cre) and B6.129P2-Lyz2tm1(cre)Ifo/J (JAX stock #004781; referred to as LyzM-cre) were crossed to B6(Cg)-Tyrc-2 J/J (JAX stock #000058; also known as B6 Albino mice) over five generations. Albino mice were chosen to reduce photon absorption by pigmented ocular structures. After the cre recombinase-expressing strains were established in the albino background, these were crossed to FVB.129S6(B6)-Gt(ROSA)26Sortm1(Luc)Kael/J (JAX stock #005125; referred to as ROSA Luc). The cre reporter strains possess a loxP-flanked STOP transcriptional sequence placed between the *luc* sequence and the *Gt(ROSA)26Sor* promoter^10^. Following Cre-mediated recombination the stop cassette is excised, allowing expression of Luciferase enzyme. The progeny of this cross, termed cre:ROSA-LUC lines, were used for the bioluminescence experiments. The genotypes used were as follows: Lck *cre/* + *; ROSA26-luciferase/* + *;Tyr (*hereafter referred to as Lck-cre:ROSA-LUC*), CD19-cre/* + *; ROSA26-luciferase/* + *;Tyr (*CD19-cre:ROSA-LUC*), LyzM-cre/* + *ROSA26-luciferase/* + *; Tyr (*LyzM-cre:ROSA-LUC*)* and *S100A8-cre/* + *; ROSA26-luciferase/* + *; Tyr* (S100A8-cre:ROSA-LUC).

Flow cytometry experiments were performed using male and female C57Bl6 mice between the ages of 6 weeks and 3 months old. Mice were maintained with standard chow and water ad libitum under specific pathogen-free conditions. Drinking water was supplemented with acetaminophen (200–300 mg/kg) post uveitis induction to minimize discomfort.

### Uveitis induction

PMU was generated as previously described^4^. Briefly, animals received a subcutaneous injection of 100 µg killed mycobacterium tuberculosis H37Ra antigen (#231141, Difco Laboratories, Detroit, MI) in 0.1 cc of an emulsion of incomplete Freund's adjuvant (#263910, Difco Laboratories, Detroit, MI). Seven days later (designated as day zero) the right eye of each animal received an intravitreal injection of 10 µg of killed mycobacterium tuberculosis H37Ra antigen in 1 µl of phosphate buffered saline (PBS). The fellow eye (left eye) of each animal is an untreated negative control. Sham injection animals received subcutaneous injection of 100 µg TB antigen followed seven days later by an intravitreal injection of PBS (1ul) into the right eye. Sham injection did not induce uveitis.

### Optical coherence tomography (OCT) image acquisition and scoring

Detection of uveitis and clinical scoring of uveitis was performed using OCT imaging^4,5^. OCT images were acquired on anesthetized animals using the Bioptigen Envisu R2300. Anesthesia was provided with 6.9 mg/kg ketamine/xylazine IP (1% solution) (Ketamine: Ketaset 100 mg/mL, Zoeitis, Inc. Kalamazoo, MI; Xylazine: AnaSed 20 mg/mL, Lloyd Laboratories, Shenandoh, IA). Eyes were dilated with phenylephrine (2.5%, Akorn, Inc. Lake Forest, IL) and corneal protection provided by Genteal (Alcon Laboratories, Inc. Fort Worth, TX). Animals were wrapped in warming gauze and placed in the prone position on the Bioptigen mouse imaging cassette. For the anterior chamber, 3.6 mm × 3.6 mm images (1000Ascan/ Bscan × 400 B-scans) were captured using a Bioptigen 12 mm telecentric lens (product # 90-BORE-G3-12, Bioptigen, Inc. Morrisville, NC). For retinal imaging, 1.6 mm × 1.6 mm images (1000A scans/ B scan × 200 B-scans) were captured using the Bioptigen mouse retina lens (product # 90-BORE-G3-M, Bioptigen, Inc. Morrisville, NC). Inflammation captured by OCT images of the anterior and posterior chambers was scored on a scale of 0 to 4 by masked graders using a system adapted from the OCT image analysis approach developed in the PMU rat model system^5^. Each image was scored by three graders and the median score designated as the final score. Score of the anterior chamber was assigned based on the following criteria: (0) for the absence of AC cell or other signs of inflammation, (0.5) for 1–5 cells in the aqueous or corneal edema, (1) for 6–20 cells in the aqueous and/or a single layer of cells across the anterior lens capsule, (2) for 20–100 cells in the aqueous or fewer than 20 cells and with a small hypopyon, (3) for 20–100 cells in the aqueous with a large hypopyon OR pupillary membrane, (4) for any number of cells in the aqueous with a large hypopyon AND pupillary membrane OR loss of anterior chamber structure detail due to severe inflammation. Score of the posterior chamber was assigned based on the following criteria: (0) for the absence of vitreous cells or other signs of inflammation, (0.5) for the presence of few vitreous cells occupying < 10% of the vitreous area and no subretinal or intraretinal infiltrates or retinal architecture changes, (1) for the presence of vitreous cells occupying between 10 and 50% of the vitreous area, and no subretinal or intraretinal infiltrates or retinal architecture changes, (2) for the presence of vitreous cells diffusely > 50% of the vitreous area and no subretinal or intraretinal infiltrates or retinal architecture changes, (3) for the presence of vitreous cells equal to grade 2 and 1 dense vitreous opacity occupying 10–20% of the vitreous area OR the presence of vitreous cells equal to grade 2 and rare (⋜ 2) subretinal or intraretinal opacities, (4) for the presence of dense vitreous opacity occupying > 20% of the vitreous area OR for the presence of vitreous cells equal to grade 2 and many (> 2) or large subretinal or intraretinal opacities. In cases of severe media opacity, posterior segment images were assigned a grade of NA when a score could not be assigned.

### In vivo bioluminescence imaging and analysis

Bioluminescence images were captured using the In Vivo Imaging System (IVIS) Spectrum (Perkin Elmer, Santa Clara, CA) and IVIS imaging software (Perkin Elmer, Santa Clara, CA). Ten minutes prior to in vivo imaging, animals received an intraperitoneal (IP) injection of D-luciferin (150 µg luciferin/kg body weight), and one drop of phenylephrine (2.5%, Akorn, Inc. Lake Forest, IL) was placed on each eye to dilate the pupil, retract the eyelids, and provide mild globe proptosis. During imaging, animals were positioned on the IVIS warming stage, continuous isoflurane anesthesia was provided by nose cone, and corneal protection provided using Genteal (Alcon Laboratories, Inc. Fort Worth, TX). Imaging was restricted to the eyes and top of the head using separation shielding bars placed above the animal covering the ears and snout. Images were acquired at stage position “A”, 1.5 cm imaging height, with medium binning and summing over a 2–5 min exposure. Length of exposure was optimized for each genotype: 5-min imaging window for Lck-cre:ROSA-LUC and CD19-cre:ROSA-LUC animals, 3-min imaging windows for LyzM-cre:ROSA-LUC animals, and 2-min imaging windows for S100A8-cre:ROSA-LUC animals. In each imaging session 3–4 sequential exposure windows were collected for each animal. Three regions of interest (ROI) were defined on each image (right eye ROI, left eye ROI, and fur ROI) and bioluminescence in flux (photons/second) was calculated using Living Image software (PerkinElmer). The mean value of the ROIs from the sequential imaging windows was determined and reported as the final value for each ROI. The mean coefficient of variation (CV) for each cre:ROSA-LUC line was calculated by first obtaining the individual CV for each ROI, then the average CVs from all days for each cre:ROSA-LUC line was determined. Eye ROIs were normalized to background bioluminescence by determining the ratio of median ocular ROI bioluminescence (right or left) to the median head (fur) ROI bioluminescence. This ratio is expressed as the RBR.

Baseline bioluminescence imaging was performed on all animals prior to uveitis induction (day-7 prior to intravitreal injection). Any animal that exhibited baseline eye ROI bioluminescence > 2 SD above or below the average for the genotype was excluded from the study due to concern for a pre-existing peri-ocular or ocular surface infectious or inflammatory condition. Bioluminescence imaging was repeated two days after intravitreal injection (day + 2) and subsequently on every seventh day (day + 7, + 14, etc.) after intravitreal injection through day 35 after intravitreal injection (day + 35).

### In vitro bioluminescence

From each bioluminescent line, spleens were collected from 3 animals, mechanically disrupted, and splenocytes were sorted into myeloid, B cell and T cell purified populations using EasySep kits: mouse CD11b positive selection (#18970), negative B cell Isolation ( #19854) and EasySep T cell Isolation Kit (#19851) respectively according to manufacturer’s instructions (all kits from Stemcell Technologies Inc., MA, USA). 1 × 10^6^ isolated cells per isolation method per animal were incubated in 300 ul DMEM media containing B-27 supplement (Life technologies), 352.5 μg/ml sodium bicarbonate, 10 mM HEPES (Life technologies), 25 U/ml penicillin, 25 μg/ml streptomycin (Life technologies), and 0.1 mM luciferin potassium salt (Biosynth) for 10 min at room temperature in an opaque 96 well plate. Then bioluminescence was determined using the GloMax Explorer Multimode Microplate Reader (Madison, WI).

### Flow cytometry

The eye was enucleated, rinsed in a flow buffer, and then placed in a 50 μl pool of flow buffer. The cornea and lens removed, and the remaining contents of the eye including the iris, vitreous, retina, retinal pigmented epithelium, and choroid were separated from the eye cup and residual extraocular tissues and placed in 50 µl of flow buffer (1 × PBS, 2% FBS). The ocular tissues were manually disrupted by cutting 4–6 times with vannas scissors. The tissue and flow buffer was then transferred to an eppendorf tube and incubated with 10 mg/mL of DNAse (Roche, Germany) and 0.5 mg/mL of collagenase (Roche, Germany) at 37 °C for 15 min. Then passed through a 70 µm filter (Fisher Scientific, Hampton,NH)and washed with flow buffer. Single cells were counted using a Nexcelom Cellometer Auto 2000 (Nexcelom Bioscience,MA), 1X 10^6^ cells per sample were stained with Zombie Aqua Fixable Viability dye (Biolegend, San Diego, CA) and thereafter incubated in FcBlock (2% per sample; Biolegend San Diego, CA). The cells were stained with primary antibodies from Biolegend, [Lys6G-AF647 (1A8,127609), Lys6C-FITC (HK1.4,128005), CD11b-PerCPCy5.5 (m1/70,101227), CD3-BV421 (17A2, 100228), CD19-BV605 (6D5, 115539), CD11c-APC/fire F780 (N418, 117351), NK1.1-PE (Pk136, 108707), CD45-BUV395 (30-F11, 565967)] for 30 min at 4 °C. After staining, cells were washed and fixed with 1% paraformaldehyde in PBS. Data was acquired with a BD LSRII flow cytometer using BD FACSDiva software (BD Bioscience, Franklin Lakes, NJ, USA). Data analysis was performed using FlowJo v10.1 software (FlowJo LLC, Ashland, OR, USA). Compensation was performed using single color controls prepared from BD Comp Beads (BD Biosciences, Franklin Lakes, NJ, USA). Cell counts were also obtained using CountBright counting beads according to manufacturer protocols. Cell lineages were defined by the following combination of cell surface markers: T cells (CD45 + ,CD3 + ,CD19-), B cells (CD45 + ,CD3-,CD19 +), natural killer (NK) cells (CD45 + ,CD3-,CD19-,cd11b + ,NK1.1 +), dendritic cells (CD45 + ,CD3-,CD19-,NK1.1-,cd11b + ,cd11c +), neutrophils (CD45 + ,CD3-,CD19-,NK1.1-, cd11c-,cd11b + ,Lys6G +), inflammatory macrophage (CD45 + ,CD3-,CD19-,NK1.1-, cd11c-,Lys6G-,cd11b + ,Lys6C +).

### Statistical analysis

Data were graphed and analyzed in GraphPad Prism (version 8.0, GraphPad Software Inc, San Diego, CA). ROIs of bioluminescence values in the inflamed eye (photons/s) were compared to baseline values and to fellow eyes using Wilcoxon matched-pairs signed rank test. Comparison of PMU to sham OCT scores was performed using the Mann Whitney U-test. P values < 0.05 were considered statistically significant.

## Supplementary information

Supplementary figures

Supplementary information

## References

[1] Chu CJ (2016). Multimodal analysis of ocular inflammation using the endotoxin-induced uveitis mouse model. Dis. Model. Mech..

[2] Pepple KL, Wilson L, Van Gelder RN (2018). Comparison of aqueous and vitreous lymphocyte populations from two rat models of experimental uveitis. Invest. Ophthalmol. Vis. Sci..

[3] Liyanage SE (2016). Flow cytometric analysis of inflammatory and resident myeloid populations in mouse ocular inflammatory models. Exp. Eye Res..

[4] Gutowski MB, Wilson L, Van Gelder RN, Pepple KL (2017). In vivo bioluminescence imaging for longitudinal monitoring of inflammation in animal models of uveitis. Invest. Ophthalmol. Vis. Sci..

[5] Pepple KL, Choi WJ, Wilson L, Van Gelder RN, Wang RK (2016). Quantitative assessment of anterior segment inflammation in a rat model of uveitis using spectral-domain optical coherence tomography. Invest. Ophthalmol. Vis. Sci..

[6] Gross S (2009). Bioluminescence imaging of myeloperoxidase activity in vivo. Nat. Med..

[7] Luker KE, Luker GD (2010). Bioluminescence imaging of reporter mice for studies of infection and inflammation. Antiviral Res..

[8] Koo V, Hamilton PW, Williamson K (2006). Non-invasive in vivo imaging in small animal research. Cell. Oncol..

[9] Zinn KR (2008). Noninvasive bioluminescence imaging in small animals. ILAR J..

[10] Safran M (2003). Mouse reporter strain for noninvasive bioluminescent imaging of cells that have undergone Cre-mediated recombination. Mol. Imaging.

[11] Mezzanotte L, Root M, Karatas H, Goun EA, Löwik CW (2017). In Vivo molecular bioluminescence imaging: new tools and applications. Trends Biotechnol..

[12] Luker GD (2002). Noninvasive bioluminescence imaging of herpes simplex virus type 1 infection and therapy in living mice. J. Virol..

[13] Ezra-Elia R (2018). Can an in vivo imaging system be used to determine localization and biodistribution of AAV5-mediated gene expression following subretinal and intravitreal delivery in mice?. Exp. Eye Res..

[14] Ji X (2009). Noninvasive visualization of retinoblastoma growth and metastasis via bioluminescence imaging. Invest. Ophthalmol. Vis. Sci..

[15] Sauer B, Henderson N (1988). Site-specific DNA recombination in mammalian cells by the Cre recombinase of bacteriophage P1. Proc. Natl. Acad. Sci. USA..

[16] Rickert RC, Roes J, Rajewsky K (1997). B lymphocyte-specific, Cre-mediated mutagenesis in mice. Nucleic Acids Res..

[17] Hennet T, Hagen FK, Tabak LA, Marth JD (1995). T-cell-specific deletion of a polypeptide N-acetylgalactosaminyl-transferase gene by site-directed recombination. Proc. Natl. Acad. Sci. USA..

[18] Clausen BE, Burkhardt C, Reith W, Renkawitz R, Förster I (1999). Conditional gene targeting in macrophages and granulocytes using LysMcre mice. Transgenic Res..

[19] Wang K, Wei G, Liu D (2012). CD19: A biomarker for B cell development, lymphoma diagnosis and therapy. Exp. Hematol. Oncol..

[20] Bielory L (2000). Allergic and immunologic disorders of the eye Part I: immunology of the eye. J. Allergy Clin. Immunol..

[21] Pepple KL (2015). Primed mycobacterial uveitis (PMU): Histologic and cytokine characterization of a model of uveitis in rats. Invest. Ophthalmol. Vis. Sci..

[22] Mruthyunjaya P (2006). Efficacy of low-release-rate fluocinolone acetonide intravitreal implants to treat experimental uveitis. Arch. Ophthalmol..

[23] Cousins, S. W. T Cell Activation within Different Intraocular Compartments during Experimental Uveitis1. in 150–155 (1992). 10.1159/000429644 1730349

[24] Cheng CK, Berger AS, Pearson PA, Ashton P, Jaffe GJ (1995). Intravitreal sustained-release dexamethasone device in the treatment of experimental uveitis. Invest. Ophthalmol. Vis. Sci..

[25] Lowder C (2011). Dexamethasone intravitreal implant for noninfectious intermediate or posterior uveitis. Arch. Ophthalmol..

[26] Jaffe GJ (2006). Fluocinolone acetonide implant (Retisert) for noninfectious posterior uveitis: thirty-four–week results of a multicenter randomized clinical study. Ophthalmology.

[27] Callanan DG, Jaffe GJ, Martin DF, Pearson PA, Comstock TL (2008). Treatment of posterior uveitis with a fluocinolone acetonide implant. Arch. Ophthalmol..

[28] Jaffe GJ (1998). Intravitreal sustained-release cyclosporine in the treatment of experimental uveitis. Ophthalmology.

[29] Czupryna J, Tsourkas A (2011). Firefly luciferase and RLuc8 exhibit differential sensitivity to oxidative stress in apoptotic cells. PLoS ONE.

[30] Njus D, Baldwin TO, Hastings JW (1974). A sensitive assay for proteolytic enzymes using bacterial luciferase as a substrate. Anal. Biochem..

[31] Pham CTN (2006). Neutrophil serine proteases: specific regulators of inflammation. Nat. Rev. Immunol..

[32] Grote J (2006). Identification of poly(ADP-ribose)polymerase-1 and Ku70/Ku80 as transcriptional regulators of S100A9 gene expression. BMC Mol. Biol..

[33] Caspi RR (1986). T cell lines mediating experimental autoimmune uveoretinitis (EAU) in the rat. J. Immunol..

[34] Shao H (2006). Severe chronic experimental autoimmune uveitis (EAU) of the C57BL/6 mouse induced by adoptive transfer of IRBP1–20-specific T cells. Exp. Eye Res..

[35] Pracht K (2017). A new staining protocol for detection of murine antibody-secreting plasma cell subsets by flow cytometry. Eur. J. Immunol..

[36] Tellier J, Nutt SL (2017). Standing out from the crowd: How to identify plasma cells. Eur. J. Immunol..

[37] Smith JR, Stempel AJ, Bharadwaj A, Appukuttan B (2016). Involvement of B cells in non-infectious uveitis. Clin. Transl. Immunol..

[38] Kielczewski JL, Horai R, Jittayasothorn Y, Chan C-C, Caspi RR (2016). Tertiary lymphoid tissue forms in retinas of mice with spontaneous autoimmune uveitis and has consequences on visual function. J. Immunol..

[39] Kleinwort KJH (2016). Immunological characterization of intraocular lymphoid follicles in a spontaneous recurrent uveitis model. Invest. Ophthalmol. Vis. Sci..

[40] Epps SJ (2019). Features of ectopic lymphoid-like structures in human uveitis. Exp. Eye Res..

[41] Heng JS (2019). Comprehensive analysis of a mouse model of spontaneous uveoretinitis using single-cell RNA sequencing. Proc. Natl. Acad. Sci. USA.

