# Supplementary material for: Bioluminescence for in vivo detection of cell-type-specific inflammation in a mouse model of uveitis
Source: Sci Rep. 2020 Jul 9;10:11377. doi: 10.1038/s41598-020-68227-4 (PMC7347586; doi:10.1038/s41598-020-68227-4)
Supplement: Supplementary file 1 — Supplementary figures [file 41598_2020_68227_MOESM1_ESM.pdf]

Bioluminescence for in vivo detection of cell-type-specific inflammation in a mouse model of uveitis

Sarah John[1], Kevin Rolnick[1], Leslie Wilson[1], Silishia Wong[1], Russell N. Van Gelder [1,2,3], Kathryn L. Pepple1,*

[1]University of Washington, Department of Ophthalmology, Seattle, Washington 98104
[2]University of Washington, Department of Biological Structure, Seattle, Washington 98195
[3]University of Washington, Department of Pathology, Seattle, Washington 98195

*Corresponding author: kpepple@uw.edu

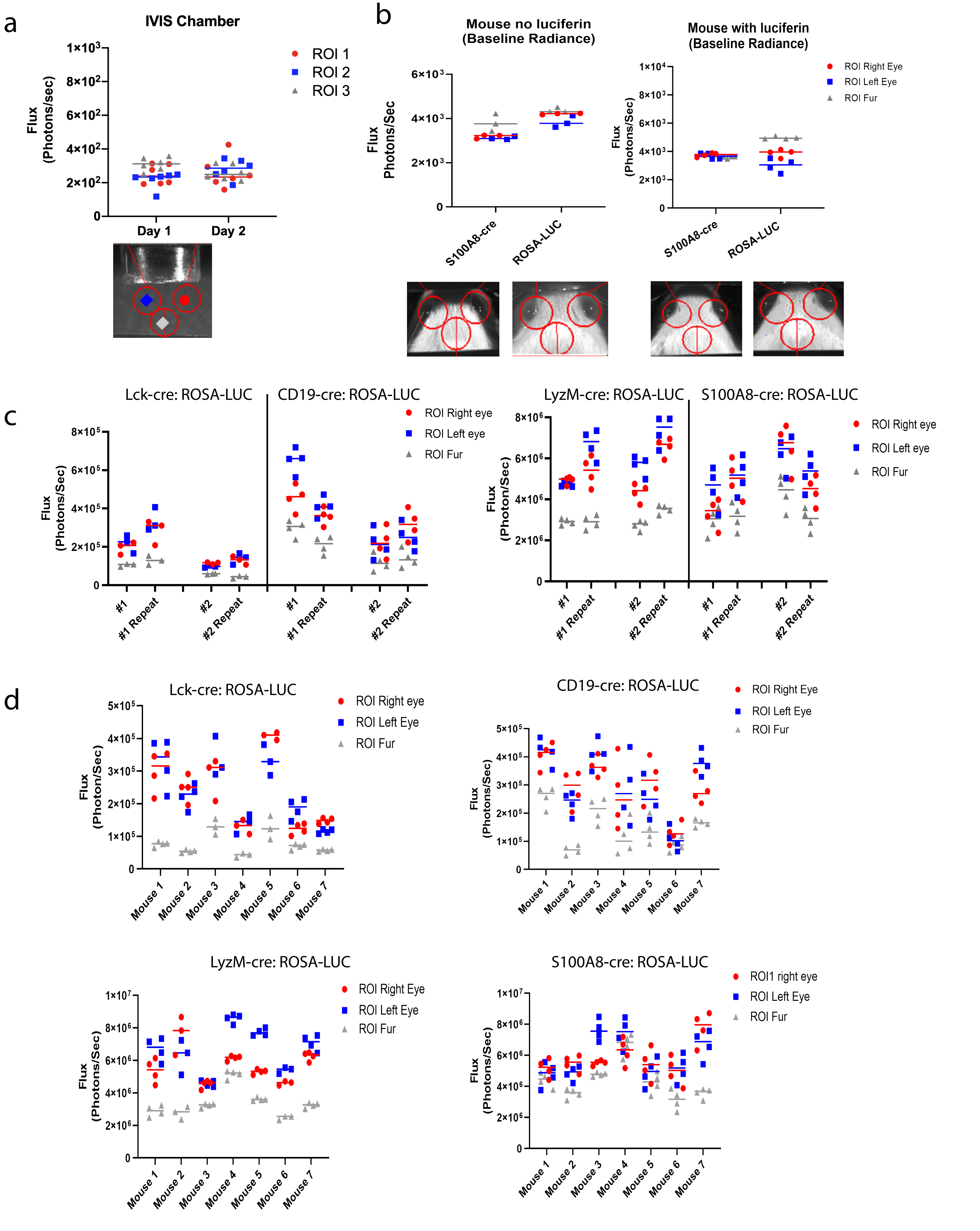

Supplementary Figure 1

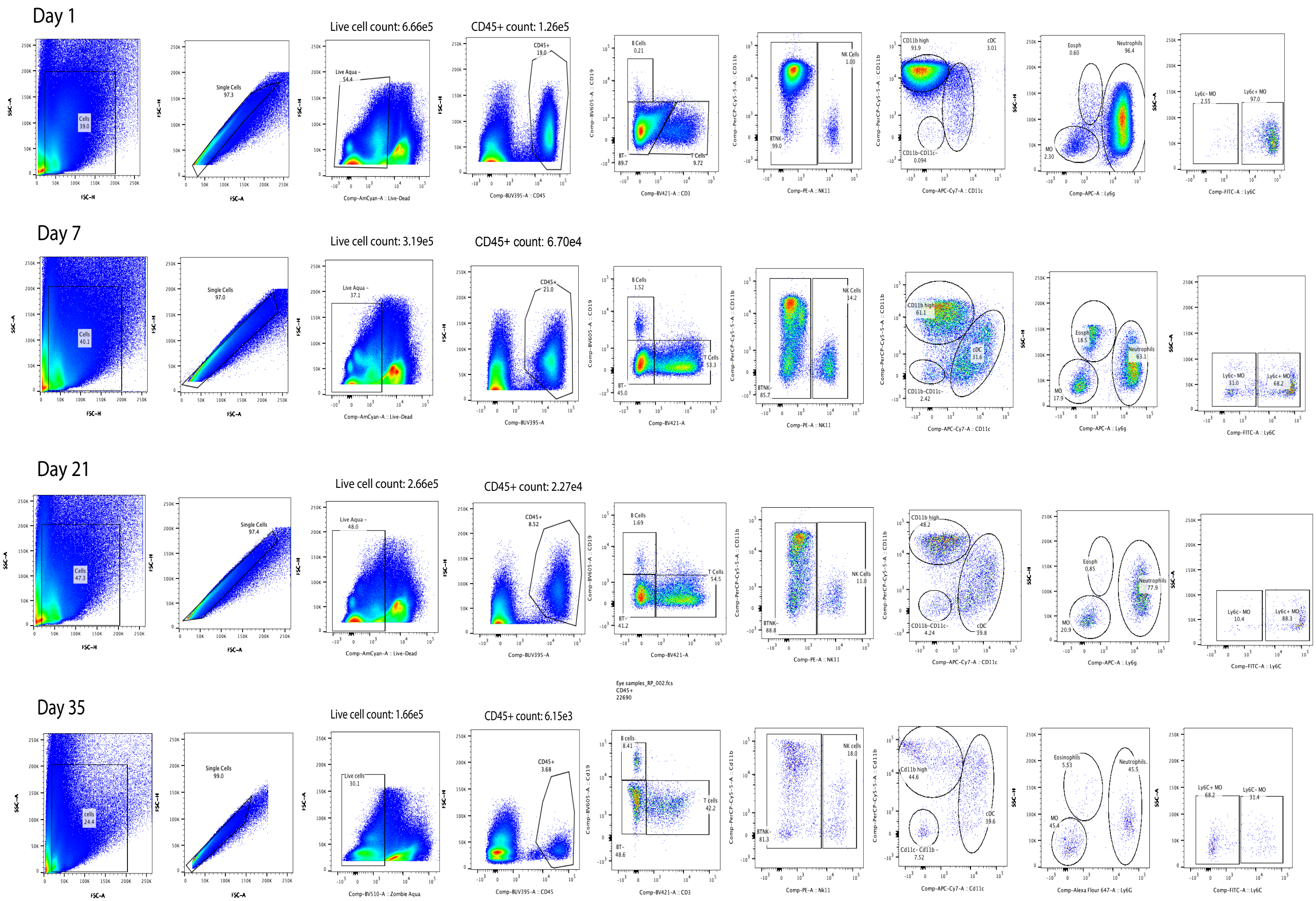

Supplementary Figure 2

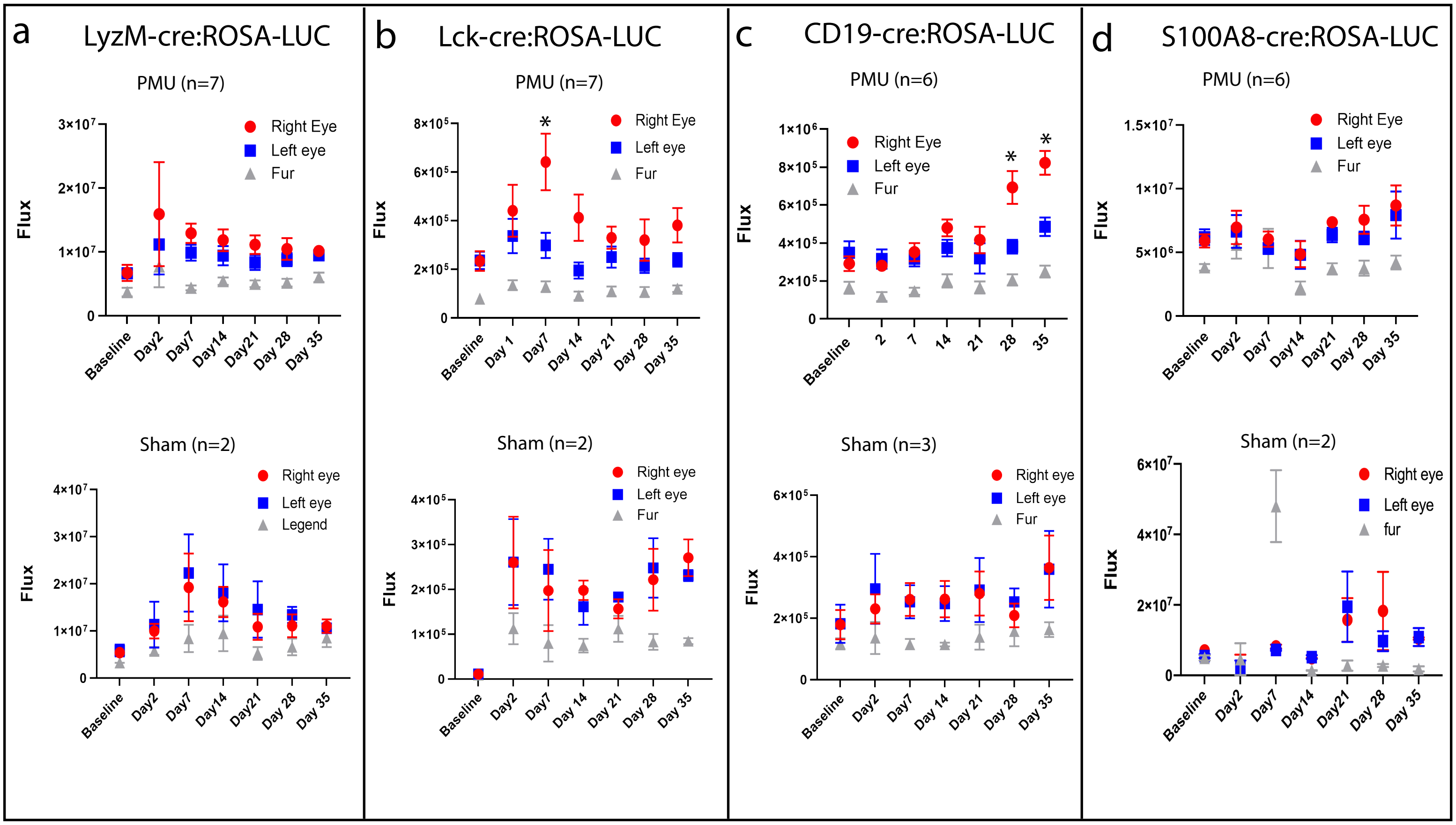

Supplementary Figure 3
