# Supplementary material for: Bioluminescence for in vivo detection of cell-type-specific inflammation in a mouse model of uveitis
Source: Sci Rep. 2020 Jul 9;10:11377. doi: 10.1038/s41598-020-68227-4 (PMC7347586; doi:10.1038/s41598-020-68227-4)
Supplement: Supplementary file 2 — Supplementary information [file 41598_2020_68227_MOESM2_ESM.docx]

**Supplementary figure 1. BLi controls. (a)** The three ROI’s used in this study for the right eye (red circle), left eye (blue square), and fur baseline (grey triangle) were used to determine the baseline bioluminescence of the IVIS chamber. In each imaging session, 3-4 images are captured, the flux from each ROI determined (individual points) and the average flux calculated for each ROI (bar). Image acquisition was performed on two separate days. The baseline bioluminescence from the chamber is in the range of 10^2^ photons/sec. **(b)** Bioluminescence in animals with either a cre- transgene or the luc transgene was measured in the absence of luciferin (left graph and images) or after IP injection of luciferin (right graph and images). **(c)** Two mice per genotype were imaged prior to initiation of uveitis on two separate days to determine the degree of variance between imaging sessions. **(d)** Baseline bioluminescence for each animal used in the longitudinal PMU study for each cell-type-specific transgenic strain.

**Supplementary figure 2. Representative flow cytometry gating strategy**. (**a)** Day 1 (**b**) Day 7 (**c**) Day 21 (**d**) Day 35. On each day all events were gated to remove debris, then for singlets, then live cells. From the singlet live cell population, CD45+ cells were selected. B cells identified from the CD19+ gate, T cells from the CD3+ gate. From the CD3-, CD19- population, NK cells were identified as the NK1.1+ population. From the CD11b+ population, dendritic cells were identified as the CD11c+ population, neutrophils as the Ly6G+ population, and macrophage as the Ly6C+ population. Final cell percentages were reported in figure 2 as the % of CD45+ cells. Percentages shown in this figure are the % of the parent gate.

**Supplementary figure 3.** **Average photon flux for PMU and Sham injection treated animals**. **(a)** Myeloid reporter LyzM-cre:ROSA-LUC **(b)** T cell reporter Lck-cre:ROSA-LUC **(c)** B cell reporter CD19-cre:ROSA-LUC **(d)** Neutrophil reporter S100A8-cre:ROSA-LUC. For each cre:ROSA-LUC line, individual animals were imaged prior to right eye injection (baseline) in the PMU and sham treated cohorts and then imaged longitudinally. On each day, the average flux from the right eye ROI (red), left eye (blue) and background/fur ROI (gray) are shown for all animals in the cohort. Average flux from right and left eye ROIs on each day were compared to baseline values using Wilcoxon matched-pairs signed rank test,* = p<0.05. In Lck-cre:ROSA-LUC the average flux from right eye ROI on day 7 was significantly higher compared to the average right eye flux on baseline (p=0.02). In CD19-cre:ROSA-LUC the average flux from right eye ROI increased significantly from baseline on days 28 (p=0.03) and 35 (p=0.02).
